# Trends in epidemiology of hyperglycemia in pregnancy in Taiwan, 2008-2017

**DOI:** 10.3389/fendo.2022.1041066

**Published:** 2023-01-06

**Authors:** Shin-Yu Lin, Yi-Ling Wu, Chun Heng Kuo, Chien-Nan Lee, Chih-Cheng Hsu, Hung-Yuan Li

**Affiliations:** ^1^ Department of Obstetrics and Gynecology, National Taiwan University Hospital, Taipei, Taiwan; ^2^ Department of Obstetrics and Gynecology, National Taiwan University College of Medicine, Taipei, Taiwan; ^3^ Institute of Population Health Sciences, National Health Research Institutes, Miaoli, Taiwan; ^4^ Department of Internal Medicine, Fu Jen Catholic University Hospital, Fu Jen Catholic University, New Taipei City, Taiwan; ^5^ School of Medicine, Fu Jen Catholic University, New Taipei City, Taiwan; ^6^ Department of Family Medicine, Min-Sheng General Hospital, Taoyuan, Taiwan; ^7^ Department of Health Services Administration, China Medical University, Taichung, Taiwan; ^8^ National Center for Geriatrics and Welfare Research, National Health Research Institutes, Miaoli, Taiwan; ^9^ Division of Endocrinology and Metabolism, Department of Internal Medicine, National Taiwan University Hospital, Taipei, Taiwan

**Keywords:** gestational diabetes mellitus, gestational hyperglycemia, pregestational diabetes, undiagnosed, maternal age

## Abstract

**Background:**

Hyperglycemia in pregnancy (HIP) increases the risk of adverse pregnancy outcomes. The increasing prevalence of overweight or obesity and the increasing proportion of pregnant women with advanced maternal age (AMA) in the recent decade may affect its prevalence. We analyzed the secular trend of HIP prevalence in 2008-2017 in Taiwan and investigated the impact of AMA in this study.

**Methods:**

This cross-sectional study used data from Health and Welfare Data Science Center. Pregnant women who registered their data in the Birth Certificate Application in 2008-2017 were recruited. Diagnosis of HIP was defined by ICD-9-CM and ICD-10-CM codes.

**Results:**

In 2008-2017, 151,306-211,768 pregnant women were recruited in different years. The proportion of women with AMA increased from 15.8% to 32.1%. Meanwhile, the prevalence increased from 0.5% to 0.9% for preexisting diabetes, 0.2% to 0.4% for undiagnosed diabetes, and 11.4% to 14.5% for GDM. Maternal age was significantly associated with the prevalence of HIP. For women aged <30 years, 30-34 years and ≥35 years, the prevalence of preexisting diabetes were 0.51%, 0.75% and 1.24%, respectively (p<0.05); the prevalence of undiagnosed diabetes were 0.18%, 0.24% and 0.37%, respectively (p<0.05); and the prevalence of GDM were 10.57%, 14.77% and 18.13%, respectively (p<0.05). In all age groups, the prevalence of HIP increased over time in 2008-2017.

**Conclusion:**

The prevalence of HIP increased in Taiwan in 2008-2017, which may result from the increasing proportion of pregnant women with AMA and the change in the diagnostic criteria for GDM.

## Introduction

Hyperglycemia in pregnancy (HIP) is an important health threat to the pregnant women and the fetus (1). It includes preexisting diabetes and gestational diabetes mellitus (GDM). In women with preexisting diabetes, diabetes is diagnosed before pregnancy; whereas in women with GDM, diabetes is diagnosed during pregnancy, usually at 24 to 28 weeks of gestation. Besides, since screening for diabetes is suggested in early pregnancy by several academic associations (2), undiagnosed diabetes mellitus may also be detected in early pregnancy for the first time. Women with HIP have higher risk of receiving Cesarean section, gestational hypertension and preeclampsia (1). Newborns delivered by women with HIP are at increased risk of macrosomia, neonatal hypoglycemia and hyperbilirubinemia, preterm delivery, birth trauma and admission to neonatal intensive care unit. In women with preexisting diabetes or undiagnosed diabetes, risk of congenital abnormalities is also higher. A recent report on the TODAY study subjects has demonstrated that women with preexisting diabetes had very high rates of maternal complications (3).

In Taiwan, the percentage of women with overweight or obesity increased gradually. A report has demonstrated that women with overweight increased from 9.7% in 2011 to 11.1% in 2016, and women with obesity increased from 5.8% in 2011 to 7.4% in 2016 (4). The increasing prevalence of overweight and obesity may increase the prevalence of HIP. Indeed, Su et al. has shown that the prevalence of GDM increased gradually from 2004 to 2015 (5). However, there is no data reported on the prevalence of preexisting diabetes and undiagnosed diabetes in Taiwan during this period of time. On the other hand, with the advance in the technologies of artificial reproduction, the increase in women’s employment, and the changes in social and cultural factors, the proportion of pregnant women with advanced maternal age (AMA, ≥35 years old) is increasing, especially in developed countries (6, 7). Women with AMA have a higher risk of GDM, as shown in our previous cohort study (8, 9). Besides, from the third decade of life, age-related glucose intolerance generally becomes more pronounced (10, 11). Theoretically, the risk of preexisting diabetes or undiagnosed diabetes may increase in women with AMA. However, data on the relationship of AMA, preexisting diabetes during pregnancy, and undiagnosed diabetes during pregnancy are limited in the literature. Taken together, these facts highlight the need for a detailed analysis on the prevalence of HIP and the impact of AMA on the prevalence of HIP.

In this study, we analyzed the change in prevalence of HIP over time in 2008-2017, including preexisting diabetes, undiagnosed diabetes, and GDM, using a nationwide database in Taiwan including 151,306-211,768 pregnant women in different years. The distribution of age in women with and without HIP were calculated, and the impact of maternal age on the prevalence in this period were investigated.

## Materials and methods

### Data source

The present study used data from Health and Welfare Data Science Center. Pregnant women whose data registered in the Birth Certificate Application in 2008-2017 were used for the analyses. The beginning of pregnancy and the 24^th^ gestational week were calculated based on the birth date of the newborn and gestational weeks at delivery which were recorded in the Birth Certificate Application. The link between the pregnant woman and her newborn was confirmed by the Birth Certificate Application and the Maternal and Child Health Database. We also linked data from the Registry for Beneficiaries such as the Ambulatory Care Expenditures by Visits and the Inpatient Expenditures by Admissions, in order to acquire information about the presence of diseases and the treatments performed.

### Research ethics approval

The study was approved by the Ethics Committee of our National Health Research Institute (NHRI IRB EC1020408-E).

### Study population

In this cross-sectional study, pregnant women who registered their data in the Birth Certificate Application in 2008-2017 were recruited. According to the diagnosis made in outpatient clinic or during admission one year before pregnancy and during pregnancy, these women were classified into four groups according to a previous publication with some modification (12), as follows:

1. Preexisting diabetes mellitus: defined by at least one diagnosis of diabetes one year before pregnancy (by International Classification of Diseases, 9th Revision, Clinical Modification (ICD-9-CM) codes 250 or International Classification of Diseases, 10th Revision, Clinical Modification (ICD-10-CM) codes E08-E13).
2. Undiagnosed diabetes mellitus: defined by at least one diagnosis of diabetes before the week 24 of the pregnancy (by ICD-9-CM codes 250 or ICD-10-CM codes E08-E13, O24.0, O24.1, O24.3 or O24.8).
3. GDM: defined by at least one diagnosis of GDM from the 24 weeks of gestation to delivery (by ICD-9-CM codes 250, 648.00-648.04 or 648.8, or ICD-10-CM codes E08-E13, O24.4, O24.9, O99.810, O99.814, O99.815).
4. Women without diabetes or GDM: pregnant women without preexisting diabetes mellitus, undiagnosed diabetes mellitus and gestational diabetes mellitus from one year before pregnancy to delivery.

### Statistical analysis

In women who got pregnant in certain year and delivered in the next year, the year of delivery was used to calculate the data. Data of gestational weeks in different subgroups were derived from the Birth Certificate Application. Categorical variables were presented as number and percentages, and continuous variables were summarized by means and standard deviations, such as age and gestational weeks. The secular trend of prevalence in different groups were analyzed by p for trend. We categorized these women into three age groups for subgroup analysis, including <30 years old, 30-34 years old and ≥35 years old. The distribution of age at delivery in women with preexisting diabetes, undiagnosed diabetes, gestational diabetes and without diabetes or gestational diabetes was tested by the chi-square test. Prevalence of preexisting diabetes, undiagnosed diabetes and gestational diabetes in different age groups were compared by chi-square test. Two-tailed testing was used for statistical significance testing, and a value of p < 0.05 was considered statistically significant. All statistical analyses were conducted with SAS software version 9.4 (SAS Institute Inc., Cary, NC).

## Results

In 2008-2017, the number of pregnancies ranged from 151,306 to 211,768 per year (**Table 1**). According to Chinese zodiac, the year 2010 was the year of Tiger, and the year 2012 was the year of Dragon. Traditionally, some women are more willing to deliver in the year of Dragon and may avoid to deliver in the year of Tiger. Excluding the highest number in 2012 and the lowest number in 2010, the number of pregnancies were between 175,302 to 195,612. During this period, the mean age at delivery increased gradually, from 29.84 years old to 31.86 years old (p<0.001). The proportion of women with AMA was also increased, from 15.8% to 32.1% (p<0.001). There was a slight change in gestational weeks at delivery during this period, and the average gestational weeks were 38.08-38.23 weeks. The comorbidity of hypertension and polycystic ovary syndrome also slightly increased from 2008 to 2017 (p for trend <0.05).

**Table 1:** Clinical characteristics and prevalence of hyperglycemia in pregnancy in pregnant women in Taiwan in 2008-2017.

	2008	2009	2010	2011	2012	2013	2014	2015	2016	2017	P for trend
**Number**	179,881	175,302	151,306	179,607	211,768	176,776	192,206	195,612	189,580	178,473	
**Age (years)**	29.84 ± 4.71	30.20 ± 4.64	30.62 ± 4.77	30.86 ± 4.63	31.03 ± 4.61	31.33 ± 4.80	31.47 ± 4.78	31.59 ± 4.88	31.75 ± 4.98	31.86 ± 5.09	<0.001
** Age ≥35 years old (N)**	28,382	29,666	30,442	38,021	46,399	44,950	50,703	55,057	57,671	57,343	
** Age ≥35 years old (%)**	15.8	16.9	20.1	21.2	21.9	25.4	26.4	28.1	30.4	32.1	<0.001
**Gestational weeks (weeks)**	38.20	38.23	38.17	38.18	38.19	38.17	38.16	38.13	38.08	38.08	<0.001
**Comorbidity (%)**											
** Hypertension**	3.3	3.5	3.6	3.4	3.5	3.8	3.5	3.8	4.1	4.6	<0.05
** Polycystic ovary syndrome**	1.1	1.2	1.4	1.0	1.1	1.1	1.2	1.5	1.6	2.0	<0.05
**Prevalence (%)**											
** Preexisting diabetes (%)**	0.5	0.7	0.8	0.7	0.7	0.8	0.9	0.9	0.9	0.9	<0.05
** Undiagnosed diabetes (%)**	0.2	0.2	0.2	0.2	0.2	0.2	0.2	0.3	0.3	0.4	<0.05
** GDM (%)**	11.4	12.6	13.3	14.0	14.1	14.7	15.2	16.0	14.7	14.5	<0.05

*Preexisting diabetes mellitus, diabetes diagnosed before pregnancy; undiagnosed diabetes, diabetes mellitus first detected in early pregnancy, before 20 weeks of gestation; GDM, gestational diabetes mellitus.

The prevalence of HIP, including preexisting diabetes, undiagnosed diabetes and GDM, increased in 2008-2017 (all p<0.05). The prevalence of preexisting diabetes increased from 0.5% in 2008 to 0.9% in 2017. The prevalence of undiagnosed diabetes was 0.2% from 2008 to 2014, and increased gradually to 0.4% in 2017. The prevalence of GDM increased gradually from 11.4% in 2008, reached a plateau of 16.0% in 2015, and then became 14.7% and 14.5% in 2016 and 2017, respectively.

In 2008-2017, the mean age at delivery increased in women with preexisting diabetes, undiagnosed diabetes and GDM and in women without HIP (**Supplementary Figure 1**). **Table 2** shows the distribution of age in different groups. For the risk of preexisting diabetes, the risk was the highest in women aged ≥40 years (OR 4.10, 95% CI 3.83-4.38), followed by women aged 35-39 years (OR 2.48, 95% CI 2.37-2.60) and women aged 30-34 years (OR 1.57, 95% CI 1.50-1.64), compared with women aged <30 years. There was a linear trend between maternal age and the risk of preexisting diabetes (p for trend <0.0001). Similarly, for undiagnosed diabetes and GDM, the risk also increased by maternal age (both p for trend<0.0001). Women aged ≥40 years had the highest risk of undiagnosed diabetes (OR 3.56, 95% CI 3.16-4.01) and GDM (OR 2.23, 95% CI 2.19-2.28), followed by women aged 35-39 years (undiagnosed diabetes OR 2.13, GDM OR 1.84) and women aged 30-34 years (undiagnosed diabetes OR 1.40, GDM OR 1.47), compared with women aged <30 years. The proportion of women aged ≥40 years were the highest in women with preexisting diabetes (8.3%) and undiagnosed diabetes (7.9%), followed by women with GDM (5.2%), and the lowest was women without HIP (3.3%). Similarly, the proportion of women aged 35-40 years were the highest in women with preexisting diabetes (29.8%) and undiagnosed diabetes (28.2%), followed by women with GDM (25.6%), and the lowest was women without HIP (19.4%). Besides, age at delivery was significantly associated with the risk of preexisting diabetes, undiagnosed diabetes, and GDM (all p for trend <0.0001). When analyzed by year (**Supplementary Figures 2**–**4**), the proportion of AMA increased over time. In women with preexisting diabetes, the proportion of women with AMA increased from 29.4% in 2008 to 45.6% in 2017 (p for trend <0.001); while the proportion of women aged <30 years decreased from 32.6% to 20.7% in this period (p for trend <0.001). In women with undiagnosed diabetes, the proportion of women with AMA increased from 25.5% to 39.0% in 2008-2017 (p for trend, 0.011). In women with GDM, the proportion of women with AMA increased from 20.4% in 2008 to 40.5% in 2017 (p for trend <0.001), and the proportion of women aged <30 years decreased from 36.7% to 22.8% (p for trend <0.001).

**Table 2:** The distribution of age at delivery in women with preexisting diabetes mellitus, undiagnosed diabetes mellitus, gestational diabetes mellitus (GDM) or without hyperglycemia in pregnancy (HIP) in Taiwan in 2008-2017.

	Preexisting diabetes			Undiagnosed diabetes			GDM			Without HIP		P value
Age at delivery	n	(%)	OR (95% CI)	n	(%)	OR (95% CI)	n	(%)	OR (95% CI)	n	(%)	
**<30 years old**	3,303	(23.1)	1.00	1,156	(25.5)*	1.00	69,006	(26.8)*	1.00	579,677	(37.3)*†‡	<0.001
**30-34 years old**	5,565	(38.9)	1.57(1.50- 1.64)	1,739	(38.3)	1.40 (1.30-1.51)	109,078	(42.3)¥∮	1.47 (1.46-1.49)	622,353	(40.0)¥₤	
**35-39 years old**	4,258	(29.8)	2.48 (2.37-2.60)	1,281	(28.2)	2.13 (1.97-2.31)	66,065	(25.6)αβ	1.84 (1.82-1.86)	301,438	(19.4)αβγ	
**≥40 years old**	1,182	(8.3)	4.10 (3.83-4.38)	359	(7.9)	3.56 (3.16-4.01)	13,449	(5.2)§∥	2.23 (2.19-2.28)	50,602	(3.3)§∥¶	
**Total**	14,308	(100.0)	P<0.0001	4,535	(100.0)	P<0.0001	257,598	(100.0)	P<0.0001	1,554,070	(100.0)	

Preexisting diabetes mellitus, diabetes diagnosed before pregnancy; undiagnosed diabetes, diabetes mellitus first detected in early pregnancy, before 20 weeks of gestation.

*p < 0.05 vs. preexisting diabetes mellitus in women <30 years old. †p < 0.05 vs. undiagnosed diabetes mellitus in women <30 years old. ‡p < 0.05 vs. GDM in women <30 years old.

¥p < 0.05 vs. preexisting diabetes mellitus in women 30-34 years old. ∮p < 0.05 vs. undiagnosed diabetes mellitus in women 30-34 years old.₤p < 0.05 vs. GDM in women 30-34 years old.

αp < 0.05 vs. preexisting diabetes mellitus in women 35-39 years old. βp < 0.05 vs. undiagnosed diabetes mellitus in women 35-39 years old.γp < 0.05 vs. GDM in women 35-39 years old.

§p < 0.05 vs. preexisting diabetes mellitus in women ≥40 years old. ∥p < 0.05 vs. undiagnosed diabetes mellitus in women ≥40 years old. ¶p < 0.05 vs. GDM in women ≥40 years old.

**Figure 1:**
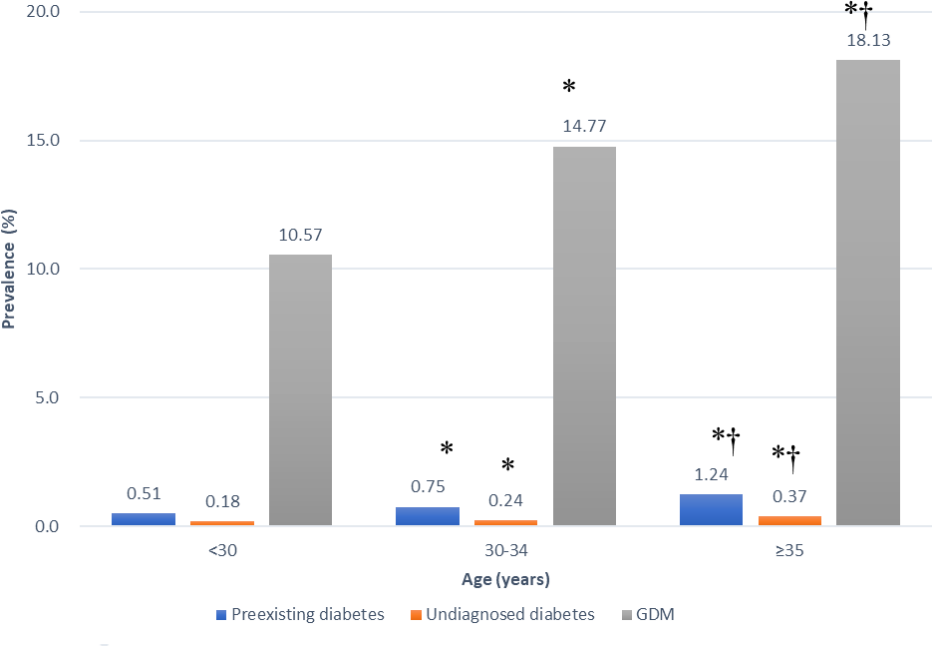
Prevalence of preexisting diabetes mellitus, undiagnosed diabetes mellitus and gestational diabetes mellitus (GDM) in different age groups in Taiwan in 2008-2017. *p < 0.05 vs. women aged <30 years. ^†^p < 0.05 vs. women aged 30-35 years.

In **Figure 1**, the prevalence of HIP increased by age. The prevalence of preexisting diabetes was the lowest in women aged < 30 years (0.51%), followed by women aged 30-34 years (0.75%, p<0.05 vs. women aged <35 years) and was the highest in women aged ≥35 years (1.24%, p<0.05 vs. women aged <30 years or women aged 30-34 years). Similar trend was observed in women with undiagnosed diabetes, the prevalence of undiagnosed diabetes in women aged <30 years, 30-34 years and ≥35 years were 0.18%, 0.24% and 0.37%, respectively (all p<0.05 comparing each other). In women with GDM, the prevalence was the lowest in women aged < 30 years (10.57%), followed by women aged 30-34 years (14.77%, p<0.05 vs. women aged <35 years) and was the highest in women aged ≥35 years (18.13%, p<0.05 vs. women aged <30 years or women aged 30-34 years). In **Table 3**, the prevalence of preexisting diabetes, undiagnosed diabetes and GDM in all age groups increased by time, from 2008 to 2017.

**Table 3:** Prevalence of preexisting diabetes mellitus, undiagnosed diabetes mellitus and gestational diabetes mellitus (GDM) in different age groups by year in Taiwan in 2008-2017*.

	2008	2009	2010	2011	2012	2013	2014	2015	2016	2017	P for trend
Preexisting diabetes (%)											
** <30 years old**	0.37	0.49	0.50	0.44	0.53	0.55	0.58	0.52	0.56	0.59	0.004
** 30-34 years old**	0.55	0.75	0.78	0.73	0.71	0.81	0.82	0.80	0.78	0.80	0.032
** ≥35 years old**	1.00	1.19	1.22	1.24	1.17	1.17	1.31	1.28	1.39	1.25	0.015
Undiagnosed diabetes (%)											
** <30 years old**	0.16	0.13	0.14	0.12	0.14	0.15	0.16	0.24	0.24	0.34	0.006
** 30-34 years old**	0.22	0.21	0.19	0.16	0.22	0.23	0.22	0.24	0.32	0.36	0.010
** ≥35 years old**	0.34	0.34	0.35	0.34	0.37	0.34	0.36	0.35	0.41	0.47	0.015
GDM (%)											
** <30 years old**	8.89	9.77	10.04	10.42	10.63	11.07	11.76	12.47	10.87	10.65	0.016
** 30-34 years old**	12.87	14.12	14.47	14.95	14.66	15.03	15.51	16.24	14.94	14.38	0.053
** ≥35 years old**	15.49	16.25	17.24	18.19	18.30	18.90	18.78	19.39	18.19	18.24	0.009

Preexisting diabetes mellitus, diabetes diagnosed before pregnancy; undiagnosed diabetes, diabetes mellitus first detected in early pregnancy, before 20 weeks of gestation.

*Prevalence was calculated by using the number of women with the indicated disease in the age group in the year as the numerator and the number of all pregnant women in the age group in the year as the denominator.

## Discussion

In the present study, we have shown that the mean age of delivery and the proportion of pregnant women with AMA increased during 2008 to 2017. In the same period of time, the prevalence of preexisting diabetes, undiagnosed diabetes, and GDM also increased, and the trend could be observed when analyzed in all population and in different age groups. The proportions of women with AMA were significantly higher in women with preexisting diabetes, undiagnosed diabetes or GDM than women without HIP. In 2008-2017, the proportions of women with AMA increase by time in women with preexisting diabetes, undiagnosed diabetes, and GDM. On the other hand, the prevalence of HIP, including preexisting diabetes, undiagnosed diabetes, and GDM, increased with advanced age.

According to the estimation by the International Diabetes Federation (IDF), the global prevalence of HIP was 16.7% in 2021, affecting 21.1 million women (13). Among them, 80.3% were women with GDM, 9.1% were women with undiagnosed diabetes and 10.6% were women with preexisting diabetes. Except for the highest prevalence of 25.9% in South-East Asia, the prevalence of HIP in other regions ranged from 13.0% in Africa to 17.2% in North America and Caribbean. In Asia, the prevalence of HIP was 14.0% by the estimation of the IDF. In the present study, the prevalence of HIP ranged from 12.2% in 2008 to 15.7% in 2017. Most pregnant women with HIP were GDM, accounting for more than 90%, and women with preexisting diabetes or undiagnosed diabetes were 4.4%-5.7% and 1.2%-2.5% of all women with HIP, respectively. For the secular trend of HIP, there are only a few reports in the literature. In a systemic review and meta-analysis for the prevalence of preexisting diabetes, the combined analysis of different studies showed that the prevalence of preexisting diabetes doubled from 0.5% to 1.0% during 1990-2020 (14), which was in concordance with the findings from the present study. For GDM, a previous report in Taiwan showed that the prevalence of GDM increased from 7.6% to 13.4% in 2004-2015 (5). Besides, two reports from Korea also demonstrated the increase in the prevalence of GDM in 2009-2011 (15) and 2012-2016 (16). Taken together, findings from the literature and the present study suggest that HIP, including preexisting diabetes, undiagnosed diabetes and GDM, is still a growing health threat for the pregnant women.

Generally, AMA is defined as age of delivery greater than 35 years (8). In the United States, the proportion of pregnant women with AMA increased from 1% in 1970s (6) to 14% in 2005 (17). In the present study, the mean age of delivery increased from 29.84 years old in 2008 to 31.86 years old in 2017, and the proportion of women with AMA increased from 14.5% to 29.6% in 2008-2017. In addition, we have shown that the prevalence of preexisting diabetes, undiagnosed diabetes, and GDM increased by age. In a meta-analysis, there is a linear relationship between maternal age and the risk GDM, and every 1-year increase in maternal age is associated with a 7.9% increase in the risk of GDM (9). Besides, age-related glucose intolerance generally becomes more pronounced in the reproductive age (10, 11), which may lead to a higher risk of preexisting diabetes during pregnancy. Taken together, these findings suggest that the increasing trend of women with AMA could be one of the reasons for the increased prevalence of HIP in 2008-2017 in the present study. Furthermore, AMA also results in increased risk of various adverse pregnancy outcomes, such as preeclampsia, intrauterine growth restriction, preterm delivery and others (8). Therefore, development of strategies to decrease the proportion of women with AMA is important to improve the health of pregnant women and their offspring. For example, education of the adverse pregnancy consequence of AMA, both to students and to young women, is a key step to lower the proportion of pregnant women with AMA. Besides, the government can make some policies to shape a friendlier environment for young women to get pregnant. In addition, researches for the treatments to lower AMA-related complications are also important and should be investigated in the future.

In the present study, there was a clinically significant increase in the prevalence of GDM, from 11.4% in 2008 to 14.5% in 2017. In the same period of time, the prevalence of preexisting diabetes and undiagnosed diabetes increased only modestly. A possible explanation is the change of the diagnosis of GDM. Currently, there are two different diagnostic criteria of GDM, the two-step method (18, 19) and the one-step method (20). Different academic associations or organizations have different suggestions, including the one-step method only, the two-step method only, or both methods (2). In the literature, the prevalence of GDM diagnosed by the one-step method is higher than that by the two-step method (21, 22). Shifting from the two-step method to the one-step method resulted in an increase in the prevalence of GDM, from 10.6% to 35.5% in a Spanish study (21), and from 2.59% to 13.44% in our previous report in Taiwan (22). Therefore, the ratio of women receiving the two-step method to the one-step method would affect the overall prevalence of GDM. In Taiwan, after the one-step method was proposed in 2010 by the IADPSG, the academic associations, including the Diabetes Association of the Republic of China (DAROC), Taiwan Association of Obstetrics and Gynecology, Taiwan Society of Perinatology, and Taiwanese Association of Diabetes Educators, have held a series of educational programs promoting the use of the one-step method, and only the one-step method was recommended in the clinical practice guideline of DAROC in 2012 (23). As a result, it is possible that more obstetricians may use the one-step method to diagnose GDM, which may be another cause for the increase in the prevalence of GDM in this study. Since 2015, the clinical practice guideline of the DAROC recommends both the one-step and the two step methods to screen GDM (24–26), which may be one potential explanation for the decline in GDM prevalence in 2016 and 2017. In addition, these educational programs may also increase the awareness of screening for undiagnosed DM and GDM for both the obstetricians/physicians and the pregnant women, which may be another reason for the increase of the prevalence of undiagnosed DM and GDM. Furthermore, according to a study in Taiwan, the percentage of women with overweight or obesity increased gradually from 2011 to 2016 (9.7%-11.1% for women with overweight, 5.8%-7.4% for women with obesity) (4). This may also contribute to the increase in the prevalence of HIP.

The strength of the study is its large sample size and the inclusion of almost all pregnant women in Taiwan in 2008-2017. Besides, we have analyzed the secular trend in the prevalence of different types of HIP, including preexisting diabetes, undiagnosed diabetes and GDM, which is rarely reported in the literature. In contrast, this study has limitations. Pregnant women without national health insurance were unrecorded and therefore not included in our study and would be misclassified as women without HIP. However, because the coverage of the national health insurance is extremely high in Taiwan (over 99%), this may comprise only a minority of patients. In addition, we cannot distinguish between one-step and two-step methods used to screen gestational diabetes in this study. Since this may be one potential reason for the increasing incidence of GDM by time observed in this study, further researches are needed to confirm the impact of screening method on the incidence of GDM in population level.

In conclusion, the prevalence of preexisting diabetes, undiagnosed diabetes and GDM increased in Taiwan in 2008-2017, which may result from the increasing proportion of pregnant women with AMA and the change in the diagnostic criteria for GDM. In the future, we should develop strategies to decrease the proportion of women with AMA, such as education programs about the adverse pregnancy consequence of AMA or policies to shape a friendlier environment for young women to get pregnant, and conduct researches to investigate novel treatments to lower AMA-related complications, including HIP, both of which are important for the reduction of the health threat of AMA to pregnant women and their offspring.

## Data availability statement

The raw data supporting the conclusions of this article will be made available by the authors, without undue reservation.

## Author contributions

S-YL wrote the manuscript and researched data. Y-LW help in the statistical analysis. CHK and C-NL researched data and contributed to discussion. C-CH and H-YL initiated the study and edited the manuscript. C-CH was responsible for the statistics and the H-YL was the clinician. C-CH and H-YL contributed equally to this paper. All authors contributed to the article and approved the submitted version.

## Supplementary material

Supplementary Figure 1 Mean age at delivery in pregnant women with preexisting diabetes mellitus (dot line), undiagnosed diabetes mellitus (short dash), gestational diabetes mellitus (long dash) or without hyperglycemia in pregnancy (HIP, solid line) in Taiwan in 2008-2017. Preexisting diabetes mellitus, diabetes diagnosed before pregnancy; Undiagnosed diabetes mellitus, diabetes mellitus first detected in early pregnancy, before 20 weeks of gestation. P for trend <0.05 for women with preexisting diabetes, women with GDM and women without HIP. P for trend=0.050 for women with undiagnosed diabetes.

Supplementary Figure 2 Distribution of age at delivery in pregnant women with preexisting diabetes mellitus in Taiwan in 2008-2017. Preexisting diabetes mellitus, diabetes diagnosed before pregnancy. P for trend over years, <0.001 for women aged <30 years, 0.030 for women aged 30-34 years and <0.001 for women aged ≥35 years.

Supplementary Figure 3 Distribution of age at delivery in pregnant women with undiagnosed diabetes mellitus in Taiwan in 2008-2017. Undiagnosed diabetes mellitus, diabetes mellitus first detected in early pregnancy, before 20 weeks of gestation. P for trend over years, 0.138 for women aged <30 years, 0.175 for women aged 30-34 years and 0.011 for women aged ≥35 years.

Supplementary Figure 4 Distribution of age at delivery in pregnant women with gestational diabetes mellitus (GDM) in Taiwan in 2008-2017. P for trend over years, <0.001 for women aged <30 years, 0.023 for women aged 30-34 years and <0.001 for women aged ≥35 years.
