# Supplementary figures and images for: Trends in epidemiology of hyperglycemia in pregnancy in Taiwan, 2008-2017
Source: Front Endocrinol (Lausanne). 2023 Jan 6;13:1041066. doi: 10.3389/fendo.2022.1041066 (PMC9852886; doi:10.3389/fendo.2022.1041066)

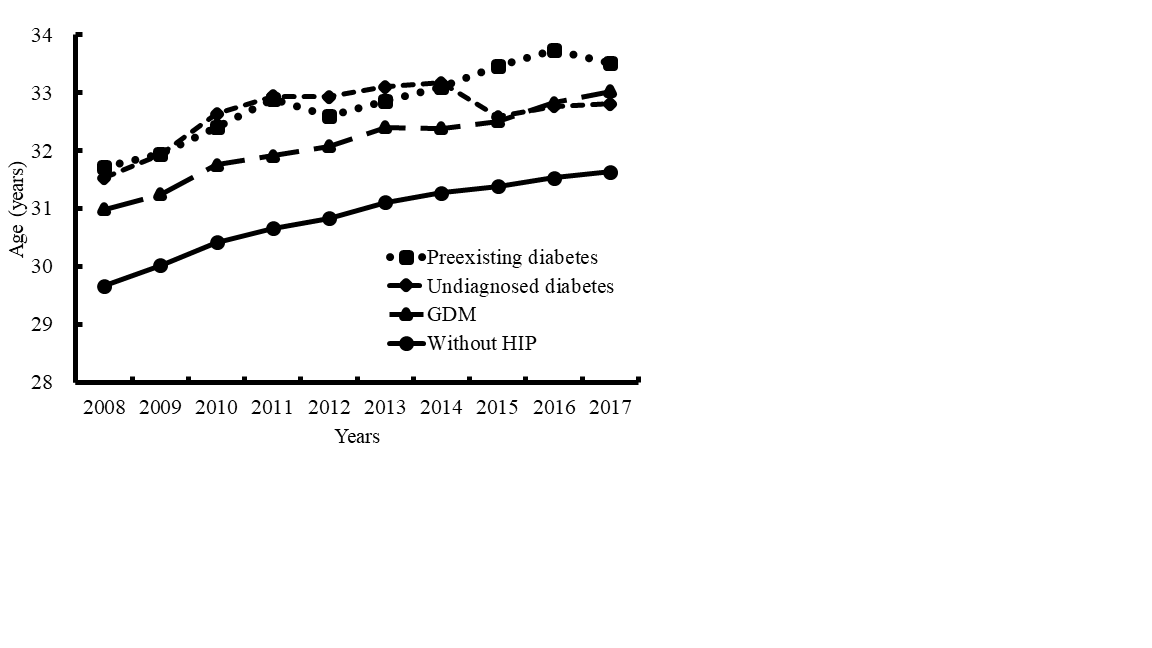

Supplement: Supplementary Figure 1 — Mean age at delivery in pregnant women with preexisting diabetes mellitus (dot line), undiagnosed diabetes mellitus (short dash), gestational diabetes mellitus (long dash) or without hyperglycemia in pregnancy (HIP, solid line) in Taiwan in 2008-2017. Preexisting diabetes mellitus, diabetes diagnosed before pregnancy; Undiagnosed diabetes mellitus, diabetes mellitus first detected in early pregnancy, before 20 weeks of gestation. P for trend <0.05 for women with preexisting diabetes, women with GDM and women without HIP. P for trend=0.050 for women with undiagnosed diabetes. [file Image_1.tif]

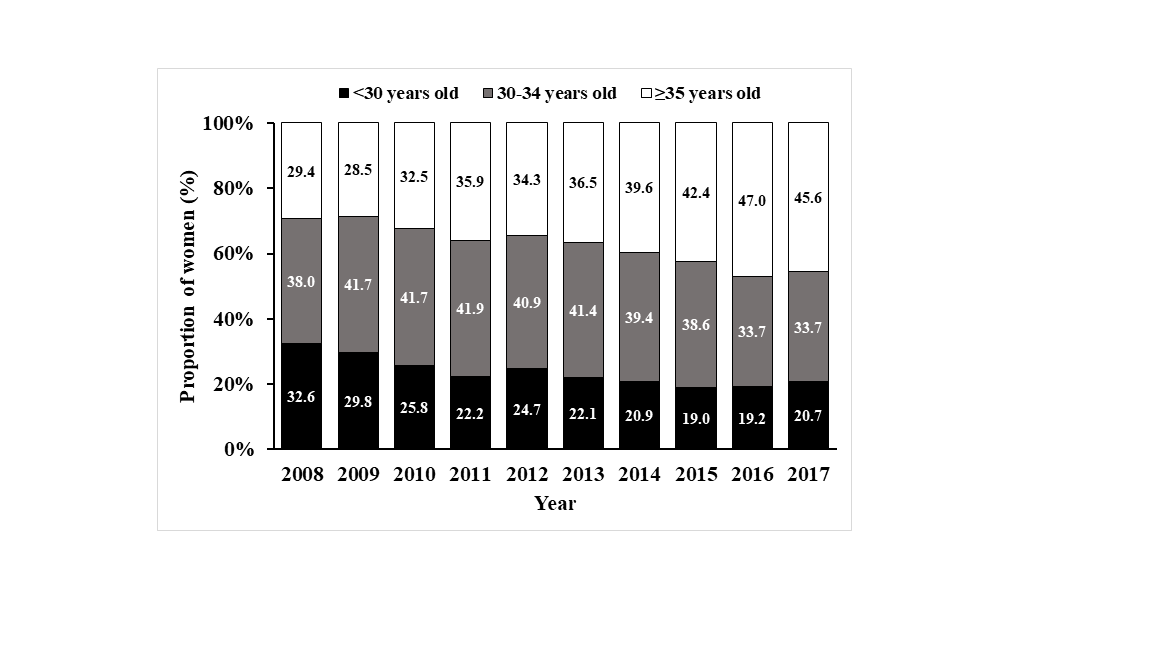

Supplement: Supplementary Figure 2 — Distribution of age at delivery in pregnant women with preexisting diabetes mellitus in Taiwan in 2008-2017. Preexisting diabetes mellitus, diabetes diagnosed before pregnancy. P for trend over years, <0.001 for women aged <30 years, 0.030 for women aged 30-34 years and <0.001 for women aged ≥35 years. [file Image_2.tif]

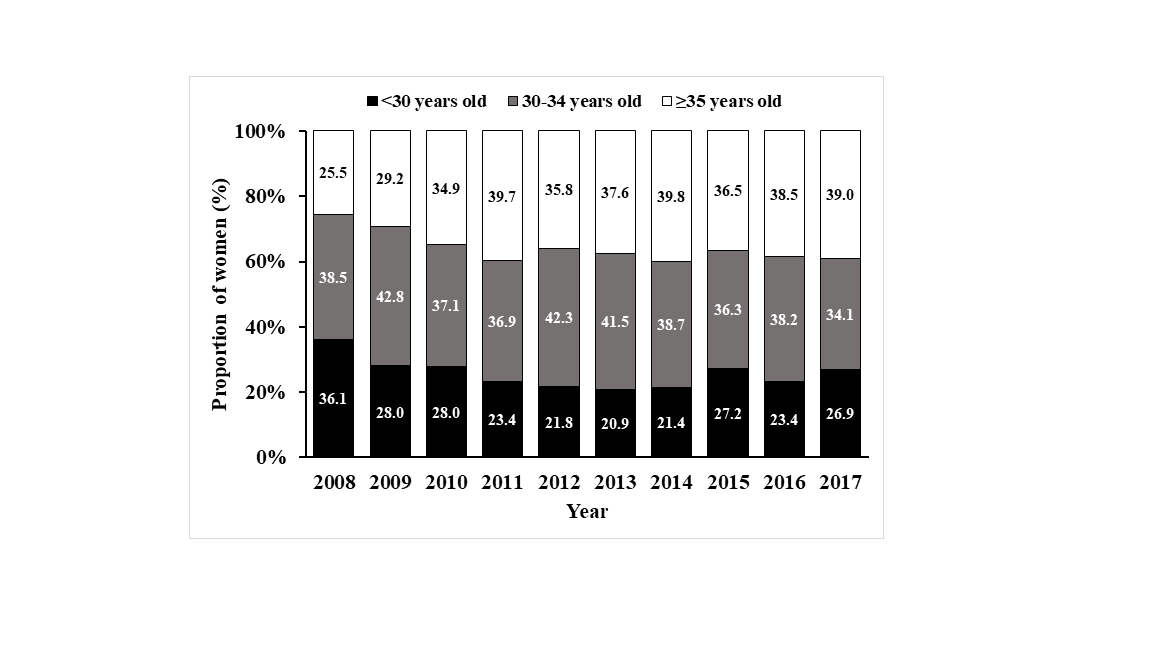

Supplement: Supplementary Figure 3 — Distribution of age at delivery in pregnant women with undiagnosed diabetes mellitus in Taiwan in 2008-2017. Undiagnosed diabetes mellitus, diabetes mellitus first detected in early pregnancy, before 20 weeks of gestation. P for trend over years, 0.138 for women aged <30 years, 0.175 for women aged 30-34 years and 0.011 for women aged ≥35 years. [file Image_3.tif]

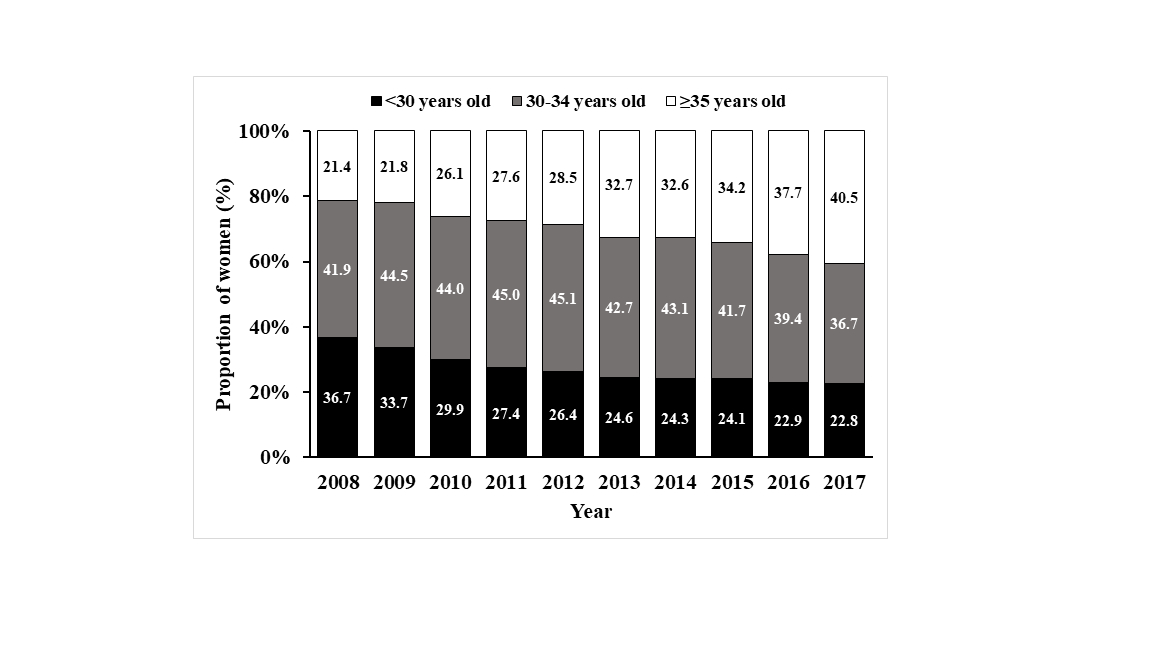

Supplement: Supplementary Figure 4 — Distribution of age at delivery in pregnant women with gestational diabetes mellitus (GDM) in Taiwan in 2008-2017. P for trend over years, <0.001 for women aged <30 years, 0.023 for women aged 30-34 years and <0.001 for women aged ≥35 years. [file Image_4.tif]
